# Neuropsychiatric symptoms in brain diseases - historical foundations

**DOI:** 10.1590/1980-57642020dn14-030014

**Published:** 2020

**Authors:** Eliasz Engelhardt, Felipe Kenji Sudo, Gilberto Sousa Alves, Jerson Laks

**Affiliations:** 1 Instituto de Neurologia Deolindo Couto; Centro de Doença de Alzheimer e outras Desordens Mentais na Velhice - Instituto de Psiquiatria, Universidade Federal do Rio de Janeiro - Rio de Janeiro, RJ, Brazil.; 2 Memory Clinic, Instituto D'Or de Ensino e Pesquisa - Rio de Janeiro, RJ, Brazil.; 3 Department of Clinical Medicine, Universidade Federal do Ceará - Fortaleza, CE, Brazil.; 4 Graduate Program in Translational Biomedicine, Universidade Grande Rio - Duque de Caxias, RJ, Brazil.

**Keywords:** behavioral symptoms, mental disorders, history, sintomas comportamentais, transtornos mentais, história

## Abstract

Neuropsychiatric symptoms, which may appear alone or combined with cognitive and neurological manifestations, are frequent in many brain dysfunctions or lesions due to vascular, traumatic, neurodegenerative, or systemic conditions. Throughout history, many of the most prominent names have contributed to the clinical definition of the currently recognized mental symptoms and syndromes. The present paper aims at providing a comprehensive overview of the development, from ancient to modern times, of some widely known concepts and constructs about such neuropsychiatric disorders.

## RESUMO.

Os sintomas neuropsiquiátricos que podem ocorrer de modo isolado ou acompanhando manifestações cognitivas e neurológicas são ocorrências frequentes em variadas disfunções ou lesões cerebrais devidas a condições vasculares, traumáticas, neurodegenerativas ou sistêmicas. Através da história, muitos dos nomes mais proeminentes contribuíram para a definição clínica dos sintomas e síndromes mentais reconhecidos atualmente. O presente artigo visa prover uma visão abrangente sobre o desenvolvimento, desde os tempos antigos até os presentes, de alguns dos conceitos e constructos amplamente reconhecidos sobre tais transtornos neuropsiquiátricos.

## INTRODUCTION

A large number of diffuse (neurodegenerative, inflammatory, biochemical, etc.), focal (vascular, traumatic, tumoral, etc.), and systemically (infectious, immunologic, metabolic, etc.)^1^ ^,^ ^2^ triggered diseases can lead to pathological brain functioning associated with a wide range of abnormal expressions, comprising, besides neurological and cognitive manifestations, several neuropsychiatric symptoms, which can be regarded as behavioral (e.g., agitation, aggressiveness) or psychological (e.g., depression, hallucinations, delusions, illusions).^2^ ^,^ ^3^ ^,^ ^4^

Such neuropsychiatric manifestations are known since the earliest times and clearly acknowledged in classical antiquity. This study will highlight some of the most frequent groups of neuropsychiatric symptoms, considered from a clinical standpoint as developed from ancient times until the modern period in Western civilizations.

In order to acquire the desired information, comprehensive research was carried out on the PubMed and Google Scholar databases, crossing neuropsychiatric symptom vs. history, in addition to a selection of references from the retrieved sources for Western classical, medieval, renaissance, and modern literature on this theme. The aim was to extract the conceptual evolution of the main groups of neuropsychiatric symptoms, including their etymological, nosological, psychopathological, and clinical aspects.

## MOOD DISORDERS

Mood disorders comprise mainly depression, mania, and bipolar disorder.^5^

### Depression and mania

Hippocrates of Kos (460-370 BC) recognized [mental] illnesses that included, besides phrenitis, the conditions of melancholia and mania, relating them to an imbalance of the four bodily humors (excess or deficiency).^6^ ‘Melancholia’, a condition identified and named by him, was attributed to an excess of black bile (from Greek *melan*=black+*kholé*=bile). He outlined the durable affective features with long-lasting feelings of fear and despair (Aphorism VI-23: “If a fright or despondency lasts for a long time, it is a melancholic affection”). The cardinal symptoms he proposed and that persisted for centuries were often associated with “aversion to food, despondency, sleeplessness, irritability, restlessness.” As for ‘mania’ (from Greek *mania*=troubling of wit, madness, fury), he described it as states of agitation related to an excess of yellow bile.^7^ ^,^ ^8^ ^,^ ^9^ ^,^ ^10^ ^,^ ^11^ Much later, Aretaeus of Cappadocia (1^st^ century AD) added the concept of ‘pneuma’ (spirit) to the humoral system. He also defined melancholia and mania; the latter as “a chronic derangement of the mind without fever,” as already mentioned by Hippocrates. Mania can be episodic and recurring, and can also occur interchangeably with melancholia, establishing a link between both (*“...*it appears that melancholia is the commencement and a part of mania”) - first conception of bipolar disorder.^10^ ^,^ ^11^ ^,^ ^12^ ^,^ ^13^ Afterward, Claudius Galenus (130‒201 AD), following the teachings of Hippocrates, agreed with the humoral doctrine and expanded this model, defining melancholia, mania, and phrenitis as ‘derangements’ of rational functions.^14^ Regarding melancholia, he argued that the gloomy symptoms were accompanied by fear and despondency and that such patients showed bizarre and fixed ideas.^15^ ^,^ ^16^ He referred to mania as a pathological excitement caused by a stinging and hot humor (yellow bile).^9^ Based on the philosophy of the tripartite soul (reason, spirit, and appetite) of Plato (429‒347 BC), he suggested that experiencing psychological stress was a potential cause for abnormality in such [mental] functions, leading to a ‘psychic imbalance.’^14^ ^,^ ^16^ ^,^ ^17^ This proposition was ignored for centuries.^6^ A detailed characterization of mood symptoms in melancholia was provided by Caelius Aurelianus (5^th^ century AD), who identified two aspects - emotional (anguish) and intellectual (delusional conception) - he described as “…mental anguish and distress, dejection, silence, animosity, sometimes a longing for death, suspicion, and crying…”.^10^ ^,^ ^15^ ^,^ ^18^ He attributed many meanings to mania, describing two kinds, as defined by Plato, “…one involving a mental strain that arises from a bodily cause of origin, the other divine or inspired…”. ^12^ The conditions of melancholia, mania, and phrenitis were considered cause for ‘loss of reason’ (i.e., ‘madness’), including variable behaviors, such as excitement, sadness (occasionally with irritability), or aggressiveness.^19^

During the subsequent centuries, medical literature on the theme was limited to variations around the humoral theory until Robert Burton (1577‒1640) published his ‘*Anatomy of Melancholy*’ (1621). Two subtypes of melancholia are described - of ‘disposition’ (transient clinical features), which corresponds to the current notion of reactions to stress or adjustment disorders, and of ‘habit’ (a more lasting or permanent condition). Burton discussed potential causes for the illness and its main components, physical and mental, some of which are still recognized as cardinal depressive symptoms.^8^ ^,^ ^20^ The humoral hypothesis was completely discarded by Phillipe Pinel (1745‒1826) in favor of a more rigorous descriptive psychopathology.^10^ He identified the symptoms of melancholia, regarded as a mental disorder, as “taciturnity, gloomy suspicion, and imaginary misfortune;” the individual could frequently be “absorbed by one exclusive idea.” He categorized melancholia into two opposite forms - one characterized by “an exalted sentiment of self-importance, associated with chimerical pretensions to unbounded power or inexhaustible riches” [mania], and another by “great depressions of spirits, pusillanimous apprehensions, and even absolute despair” [depression]. He noted that many cases of melancholia remained in such state for a long time, while others “became decided maniacs” [bipolar disorder] (1801).^10^ ^,^ ^21^ Melancholia regained its connotation of sadness, distinct from manic states, in studies published during the following years. A more detailed explanation was offered by Johann Christoph August Heinroth (1773‒1843), who reported that soul [mind] disorders (*Seelenstörungen*) might have an internal element (predisposing factor) he called the ‘soul's mood’ (*Seelenstimmung*), as well as a stimulus(i) (*Reiz, Reize*), which could be external or internal, with a ‘positive’ or ‘negative’ reaction of the soul that would result in soul disorders - ‘exaltation’ or ‘depression’ (from the Latin *deprimere=*to press down), related to ‘mania’ and ‘melancholia’, respectively, besides mixed forms. The adoption of the term ‘depression’, encompassing melancholia, stems from his work (1818).^15^ ^,^ ^22^ In parallel, Jean-Étienne Dominique Esquirol (1772‒1840) stated that melancholia was “characterized by moroseness, fear, and prolonged sadness,” and proposed the term ‘monomania’ to express a circumscribed mental disturbance, which yielded a “partial, permanent, gay, or sad” delusion. He defined the ‘sad’ type as ‘lypemania’ (from the Greek *lype*=grief, sorrow) and the gay as the ‘expansive’ type (monomania proper).^9^ ^,^ ^23^ Lastly, Emil Kraepelin (1856‒1926) published a system of psychological disorders centered on symptom patterns (syndromes) suggestive of an underlying physiological cause (1883). He classified mental disorders into various categories, which varied among the editions of his book (1881‒1913), which comprised mood disorders, including mania, melancholia, and depression, among others (1896).^8^ ^,^ ^15^ ^,^ ^24^ ^,^ ^25^ He also grouped all abnormal mood states (melancholia and mania) within the unique nosology of ‘manic-depressive psychoses’ [bipolar disorder], believing that they represented a single morbid condition.^8^ ^,^ ^15^ ^,^ ^24^ ^,^ ^25^ ^,^ ^26^

 The wide notion of ‘melancholia’ was gradually replaced by the narrower and more specific concept of ‘depression.’ The broad concept of ‘mania’ acquired a stricter meaning, maintaining the original denomination. Simultaneously, the notion of bipolar disorder arose.

## PSYCHOTIC SYMPTOMS

Psychotic symptoms include delusions, hallucinations, and illusions.^5^

### Delusions

The first descriptions of ‘delirious states’ (consisting of delirium and delusions) date from ancient times under the concept of ‘phrenitis’, one of the disease categories described by Hippocrates, featuring mental abnormalities produced by fever, poisoning, or head trauma, caused by humoral imbalance (usually due to bile). The [cumulative] symptoms include fever, derangements of the mind (rational faculties), loss of reason, wandering, unfocused speech, with the delirious state remaining throughout the illness, followed at the end by sleepiness (or insomnia, awake, and restless states), stupor, coma, and convulsions. Finally, most patients progress to death. The term continued to be commonly, when it became listed under mental disorders such as ‘delirium’, ‘clouding’, or ‘confusion’.^19^ ^,^ ^27^ The concept remained until Galenus' time, and lasted until the 19^th^ century.^19^ ^,^ ^27^ Galenus' view was based upon the traditional one - he identified phrenitis as a primary affection of the mind, capable of damaging the powers of sensation and rational faculty, both powers regarded as brain activities.^19^ The term ‘delirium’ (from the Latin *deliratio, delirationis* meaning *deliro*=‘when a plowman goes out of the furrow’ or ‘goes not right’) was introduced by Celsus (1^st^ century AD) to define mental disorders during fever or head trauma, in the same way as Hippocrates.^11^ ^,^ ^28^

The subject began to be better clarified by William Cullen (1710‒1790), who used the term ‘delirium’ to refer to errors in judgment of all sorts. He identified two types of manifestations, those associated with comatose states or fever (pyrexia) [delirium], and those not related to such conditions; the latter being called ‘insanity’ (*vesaniae*) (madness, insanity, frenzy) [delusion] (1793).^29^ Later, Esquirol defined: “A man is in delirium when his sensations are not at all in agreement with external objects, when his ideas are not at all in agreement with his sensations, when his judgments and his resolutions are not at all in agreement with his ideas, when his ideas, judgements and resolutions are independent of his volition” (1845).^23^ Thus, it was understood as the designation of incongruence among sensations, ideas, judgments, and decisions. A definitive separation between what is now accepted as ‘delusion’ and ‘delirium’, as suggested by Cullen, was motivated by Philippe Chaslin (1857‒1923), who proposed the denomination of ‘confusion’ as incoordination and slowing of ideas, distinguished from other acute conditions he named as ‘primitive mental confusion’ (*confusion mentale primitive*), split into two forms - idiopathic and secondary (associated with infectious diseases, dementia, infarction, etc.) (1890).^30^

Therefore, delirium and delusion stem from common ancestry, the ancient concept of ‘phrenitis’, with their separation beginning in the 18^th^ century.

### Hallucinations

The term was employed by the Roman statesman Marcus Tulius Cicero (106‒43 BC) to designate “the intent to mislead or equivocate” (from the Latin *allucinor* or *allucinaris* or *allucinare*=to deceive, to beguile himself, to erre) [hallucination].^11^ ^,^ ^31^ A transcendent perspective was adopted by Augustine of Hippo (354‒430), who tried to characterize ‘visions’ as mystical experiences, and emphasized the deceitful nature of visions and hallucinations, claiming that they consisted of impressions of non-existing objects produced by the skeptical soul, which could threaten one’s faith on the truth brought by the senses.^31^ He was followed later by Thomas Aquinas (1225‒1274), who established a difference between normal and false perceptions, arguing that a ‘vision’ (*visio*) was a normal phenomenon brought about by God or the devil.^31^

The term ‘hallucination’ was introduced to the psychiatric glossary by Esquirol, and, consistent with Augustine’s idea of the lack of one’s awareness of the false nature of the phenomena, he defined: “A person is said to labor under a hallucination… who has a thorough conviction of the perception of a sensation, when no external object… has impressed the senses.”^23^ ^,^ ^31^ Originally, it had been described as a visual-perceptive disturbance, but soon Esquirol expanded this concept to encompass abnormalities of single or combined sensory modalities. Importantly, he explained that “hallucinations occur in the sensory center of the brain,” not in the extremities of sense organs.^23^ ^,^ ^31^ ^,^ ^32^

### Illusions

The term ‘illusion’ (from the Latin *illusio, illusionis*=derision, scorn, deception), a highly frequent sensory-perceptual manifestation in mental disorders, was also apparently derived from Cicero’s works.^11^ ^,^ ^31^

Esquirol explained that illusions, “so frequent among the insane, deceive them respecting the qualities, relations and causes of the impressions actually received, and cause them to form false judgments respecting their internal and external sensations” (1845).^23^ ^,^ ^33^ With this definition, authors have since characterized illusions as “distorted perceptions of real stimuli”.^31^

These conditions that form the psychotic core, hallucinations, delusions, and illusions began to be delineated in ancient times. In parallel, the notion of delirium emerged.

## CHALLENGING BEHAVIORS

Challenging behaviors include agitation and aggression.^5^

### Agitation and aggression

According to Hippocrates agitation and aggressive behavior were both mediated by humoral imbalance (excessive yellow bile), which generated ‘wrath’ (rage, fury).^34^ Later, Galenus endorsed and expanded such view with the concept of ‘rage’ or ‘anger’ (Greek=*thumos*), terms already used, among others, in Homeros's writings (8^th^ century BC), expressed in words (threats, insults, indignant complaints) and deeds (violence).^35^ He related this behavior to the emotional state and physical responses and defined “…rage is not simply an increase, but as it were a kind of boiling of the hot in the heart…”.^36^ Such behaviors frequently accompany other mental disorders (‘madness’) (e.g., mania, delusion, etc.), as seen above.^19^

More recently, Esquirol denominated ‘fury’ as a reaction that could occur in the insane. He described the condition as: “…a violent excitement caused by an error of mind or heart…” (1845). He called ‘furious’ the one who “…transported by delirium [delusion], or some passion, exhausts himself by talk, by threats and actions, seeking at the same time to injure others and himself…”. According to him, fury represented one of the most alarming symptoms of insanity.^23^

Thus, challenging behaviors - agitation and aggression - are known for a long time, appearing with considerable frequency, observed alone or associated with other symptoms.

Some limitations of this review ought to be exposed. Integrating into symptomatic clusters such complex classical descriptions or centuries-distant pieces of literature can be challenging. The inherent constraints of interpreting historical concepts from a modern scientific perspective might be significant, with the risk of resulting in biased or reductive points-of-view. Finally, numerous authors might not have been included in this review due to scarce published data or the choice to keep the focus on the most relevant literature on the theme. Other neuropsychiatric manifestations, such as ‘anxiety’, ‘apathy’, ‘eating disorders’, ‘sexual disorders’, and so forth, associated with equally relevant brain conditions should and will be appraised in another opportunity.

## CONCLUSION

Neuropsychiatric (behavioral and psychological) symptoms, which frequently appear in many brain diseases, are presently seen as well-established knowledge. Such concepts have their foundations clearly rooted in very ancient teachings, when their bases were laid, evolving slowly and laboriously over millennia, with the contribution of numerous and diversely accredited authors, in order to reach the current understanding.
